# Utilizing machine learning algorithms to identify biomarkers associated with diabetic nephropathy: A review

**DOI:** 10.1097/MD.0000000000037235

**Published:** 2024-02-23

**Authors:** Baihan Dong, Xiaona Liu, Siming Yu

**Affiliations:** a The First Affiliated Hospital of Heilongjiang University of Chinese Medicine, Harbin, Heilongjiang Province, China; b Binzhou Hospital of Chinese Medicine, Binzhou, Shandong Province, China.

**Keywords:** biomarker, diabetic nephropathy, machine learning, WGCNA

## Abstract

Diabetic nephropathy (DN), a multifaceted disease with various contributing factors, presents challenges in understanding its underlying causes. Uncovering biomarkers linked to this condition can shed light on its pathogenesis and support the creation of new diagnostic and treatment methods. Gene expression data were sourced from accessible public databases, and Weighted Gene Co-expression Network Analysis (WGCNA)was employed to pinpoint gene co-expression modules relevant to DN. Subsequently, various machine learning techniques, such as random forest, lasso regression algorithm (LASSO), and support vector machine-recursive feature elimination (SVM-REF), were utilized for distinguishing DN cases from controls using the identified gene modules. Additionally, functional enrichment analyses were conducted to explore the biological roles of these genes. Our analysis revealed 131 genes showing distinct expression patterns between controlled and uncontrolled groups. During the integrated WCGNA, we identified 61 co-expressed genes encompassing both categories. The enrichment analysis highlighted involvement in various immune responses and complex activities. Techniques like Random Forest, LASSO, and SVM-REF were applied to pinpoint key hub genes, leading to the identification of VWF and DNASE1L3. In the context of DN, they demonstrated significant consistency in both expression and function. Our research uncovered potential biomarkers for DN through the application of WGCNA and various machine learning methods. The results indicate that 2 central genes could serve as innovative diagnostic indicators and therapeutic targets for this disease. This discovery offers fresh perspectives on the development of DN and could contribute to the advancement of new diagnostic and treatment approaches.

## 1. Introduction

Diabetic nephropathy (DN), a common and severe complication of diabetes mellitus (DM), ranks as one of the leading causes of end-stage renal failure globally. It is also associated with increased morbidity and mortality rates among diabetic patients. Currently, over half a billion people worldwide suffer from DM,^[1]^ indicating that more than 10.5% of the global adult population is affected by this condition. The prevalence of DM is similar in both men and women, with the highest rates observed in individuals aged 75-79. It is projected that by 2045, the global prevalence of DM in the 20-79 age group will rise to 12.2%, affecting an estimated 783 million people.^[2]^

The pathogenesis of DN is intricate and not yet fully understood, leading to suboptimal treatment outcomes.^[3]^ Standard treatments, which primarily focus on strict blood sugar and blood pressure control, have proven inadequate in halting the progression of DN to end-stage renal disease^[4]^ and in reducing DN-associated mortality rates.^[5]^ The development of DN involves multiple pathways and mediators, such as oxidative stress and inflammatory processes, which have recently been recognized as playing a significant role.^[6]^ Standard management strategies for DN, including tight glucose and blood pressure control and blocking the Renin-Angiotensin-Aldosterone System (RAAS),^[7]^ merely slow down the disease progression and are unable to stop or reverse it, often leading to end-stage renal disease in many diabetic patients. Current therapeutic approaches include anti-inflammatory^[8]^ and antioxidant treatments,^[9]^ along with the use of mineralocorticoid receptor antagonists, endothelin receptor antagonists, and vitamin D receptor activators.^[10]^ Epigenetics is increasingly acknowledged as crucial in understanding the pathogenesis and progression of DN.^[11]^ With advancements in medical technology, significant progress has been made in diagnosing and treating DN. Identifying key characteristics of genes involved in DN onset and progression can reveal new potential targets, aiding in the development of innovative treatment strategies.

This study objective was to identify crucial genes linked to DK, enhance the screening of diagnostic biomarkers in a more thorough, efficient, and precise manner, and lay a foundation for comprehending DN mechanism. Such insights could be vital for improving the diagnosis and treatment of DN.

## 2. Data and methods

### 2.1. Design and methods

**Figure 1.**
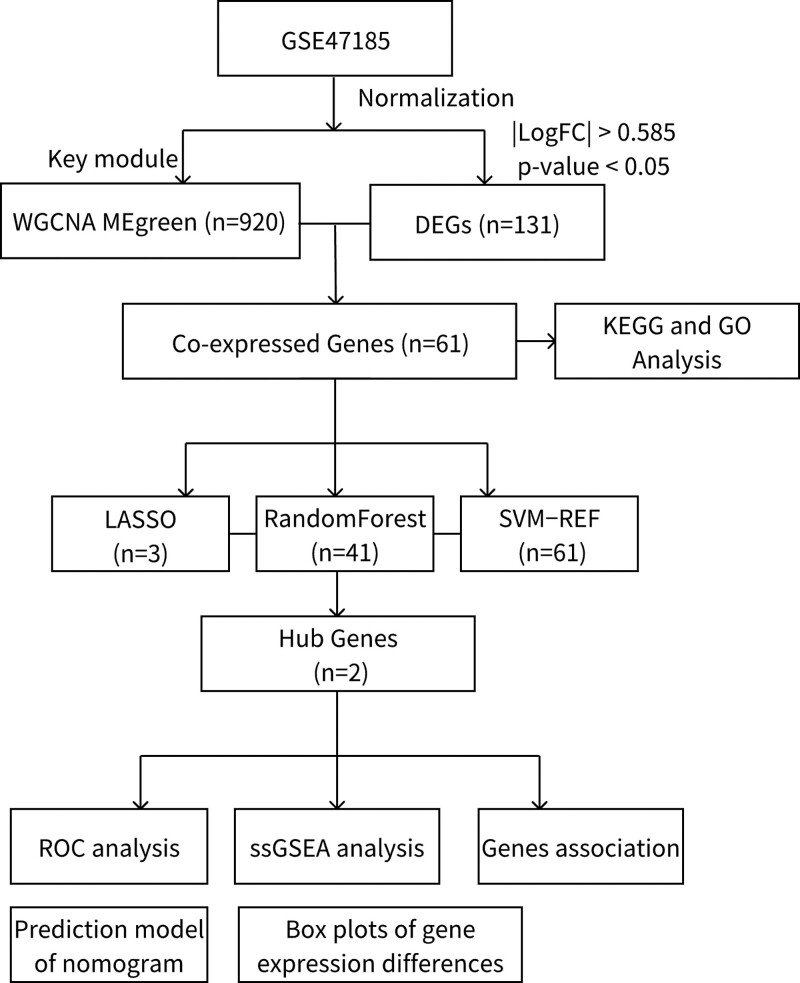
Flowchart.

Three machine learning algorithms were applied to analyze gene expression profiles in DN, using whole transcriptome sequencing. We selected intersecting differential genes from various databases and identified key genes linked to DN. These potential biomarkers then underwent enrichment analysis and validation. Figure 1 illustrates the workflow chart of data preparation, processing, analysis, and validation.

### 2.2. Data acquisition

Using bioinformatics and systems biology, we explored the common morbidity and genetic links of DN. We analyzed microarray and RNA-Seq data from the NCBI GEO database (https://www.ncbi.nlm.nih.gov/geo). Specifically, we collected a raw dataset of human gene expression related to DN, identified as GSE47185, on the GPL14663 platform (Affymetrix Human Genome U133 Plus 2.0 Arra). In this set of data, we selected 4 of these samples as the control group and 11 DN samples as the experimental group.

### 2.3. Identification of DEGs

We used R software (version 4.3.1) to identify differentially expressed genes (DEGs) in serum samples from DN and non-DN. Given the study limited sample size, we employed a T-test with a random variance model correction to identify DEGs. Genes meeting the criteria of an adjusted *P*-value of 0.05 or less and a log2 |fold change| of 0.585 or higher were classified as DEGs.

### 2.4. WGCNA analysis

To identify significant genes, we selected key modules based on the correlation between module members. Using the Weighted Gene Co-expression Network Analysis (WGCNA) package in R, we screened for hub genes.^[12]^

WGCNA is a robust method for identifying and summarizing clusters of highly correlated genes, known as modules, often using the module eigengene or an intramodular hub gene. This approach is invaluable for uncovering the biological significance of these gene clusters, particularly their relationships with specific traits or diseases. WGCNA simplifies the interpretation of complex genomic data and is notably resilient to noise. However, it is computationally demanding, especially when handling large datasets. The method reliance on setting arbitrary thresholds and its assumption of linear gene relationships can potentially lead to oversights in complex interactions. Additionally, interpreting the biological relevance of these modules can be challenging, heavily relying on the quality of the data. WGCNA also employs eigengene network methodology to explore the relationships between gene modules and external sample traits, calculating module membership measures. This facilitates network-based gene screening, aiding in the identification of potential biomarkers or therapeutic targets.

Initially, we calculated gene correlations to construct a Topological Overlap Matrix (TOM). We then calculated the dissimilarity TOM (diss TOM = 1 - TOM) and established a phylogenetic clustering tree based on the hierarchical clustering of diss TOM. To classify genes with similar expression profiles into gene modules, the average linkage hierarchical clustering of the gene tree was performed using a “TOMbased” difference measurement method with a minimum genome size of 10. 0.25 was considered a clustering height restriction, and the module membership and gene significance were calculated. Genes from these key modules were then selected for further analysis.

### 2.5. Enrichment analysis

Enrichment analysis is a widely used method in bioinformatics for identifying overrepresented biological themes within gene sets. One of its main advantages is the ability to provide insights into the biological processes (BP), pathways, or other categories that are significantly associated with a list of genes. This is particularly useful in interpreting large-scale genomic data, helping researchers understand the functional implications of their experiments.

However, enrichment analysis has some limitations. One key issue is its dependency on the quality and completeness of the annotation databases it uses, which can impact the accuracy of the results. Additionally, the method may produce false positives due to the multiple testing inherent in examining large sets of genes against numerous categories. There also a challenge in interpreting the results, as significant enrichment does not always imply a direct biological connection.

We will select genes from their corresponding modules and intersect with previously obtained DEGs to obtain and co-express genes. We conducted Kyoto Encyclopedia of Genes and Genomes (KEGG) and Gene Ontology (GO) enrichment analyses on the identified co-express genes using R software.

### 2.6. Hub genes

To identify candidate genes, we utilized 3 distinct methods in Random Forest, LASSO, and SVM-RFE to further pinpoint hub genes from co-express genes, which were those identified by all 3 methods.^[13]^

SVM-RFE, LASSO, and Random Forest each offer distinct advantages in machine learning. SVM-RFE excels in feature selection and reducing overfitting, especially in high-dimensional data. LASSO is notable for feature selection and regularization, encouraging sparse solutions and preventing overfitting. Random Forest is known for its accuracy with large datasets and robustness in handling unbalanced data, offering insights into feature importance. Together, these methods enhance machine learning models’ predictive power and efficiency, each contributing unique strengths across various data scenarios. Additionally, while LASSO is easy to interpret but may struggle with complex features, SVM-RFE is versatile but less scalable, and Random Forest is excellent for feature importance but complex in tree construction. This multifaceted approach leverages each method advantages, ensuring robust results.

### 2.7. Hub genes verification

We verified the hub genes by assessing their diagnostic efficacy through receiver operating characteristic (ROC) curves and examining their expression profiles in the dataset. ROC curves were plotted using ROC packages in R, and the area under these curves (AUC) was calculated. The closer the AUC value is to 1, the more accurate the prediction is. Of course, *P*-value needs to be below 0.05 to confirm statistical significance.^[14]^ Genes meeting these criteria were established as reliable diagnostic biomarkers for DN.

### 2.8. ssGSEA of hub genes

ssGSEA (single-sample Gene Set Enrichment Analysis) is a method used to assess the presence and activity of specific gene sets in individual samples, particularly in transcriptomic data. It ranks genes by expression levels and calculates enrichment scores for gene sets, aiding in identifying biomarkers and understanding biological pathways in disease and treatment studies. We conducted ssGSEA analysis on key hub genes to predict their expression levels.

## 3. Results

### 3.1. Screening of DEGs

**Figure 2.**
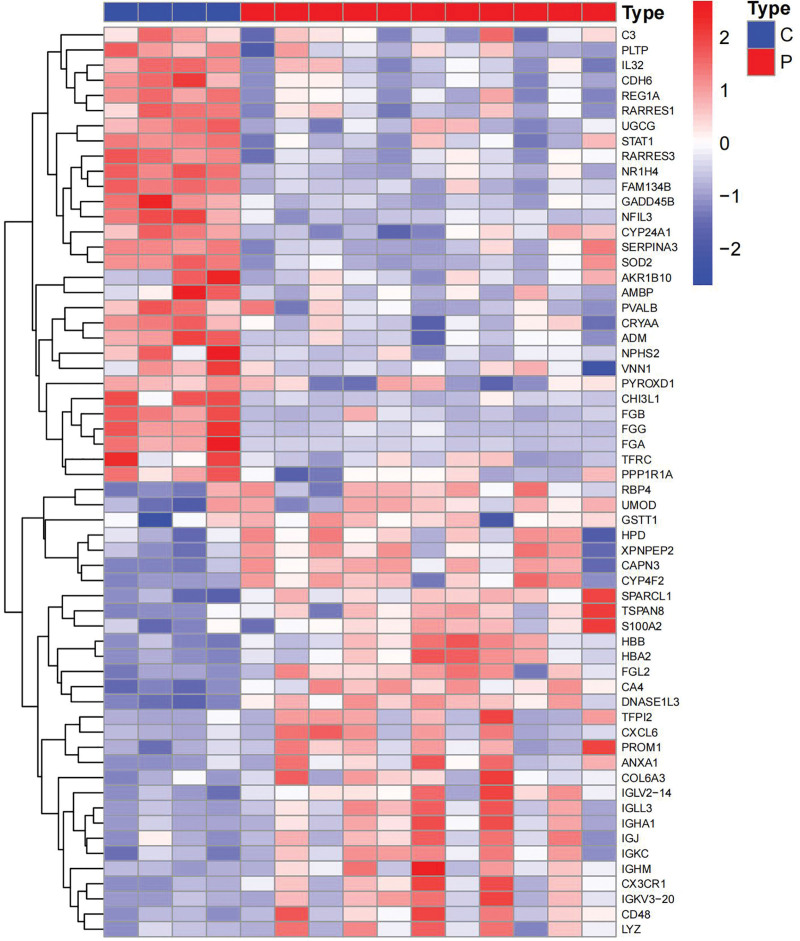
DEGs heatmap. DEG = differentially expressed gene.

**Figure 3.**
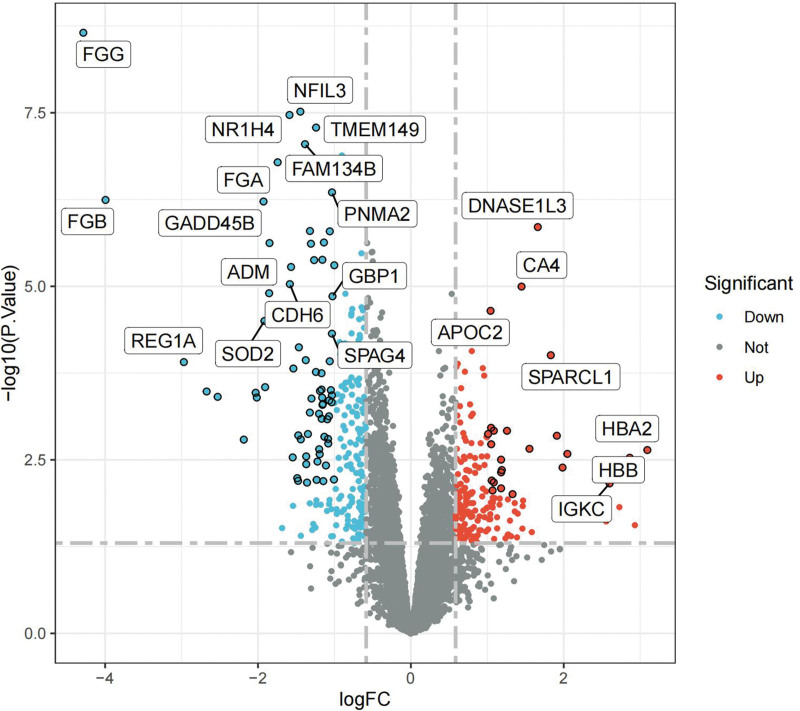
DEGs volcano plot. DEG = differentially expressed gene.

Following our specified screening criteria, we identified a total of 131 differentially expressed genes (DEGs) in both controlled and uncontrolled samples (Table S1, http://links.lww.com/MD/L699). Of these, 112 genes were found to be downregulated and 19 upregulated. The distribution and characteristics of these DEGs are illustrated in a heat map and a volcano plot (Figs. 2 and 3) respectively.

### 3.2. WGCNA and co-express genes

**Figure 4.**
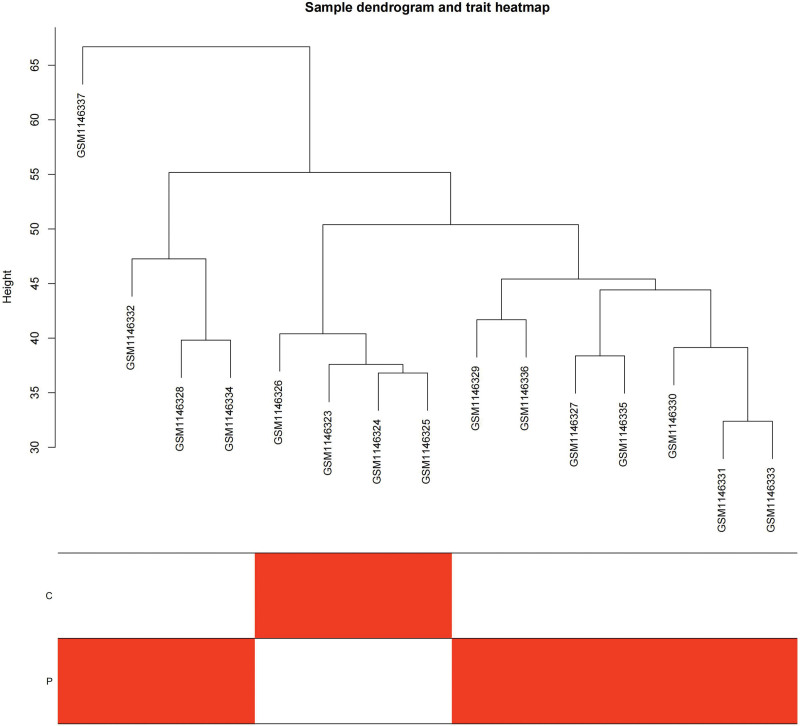
Sample dendrogram and trait heatmap.

**Figure 5.**
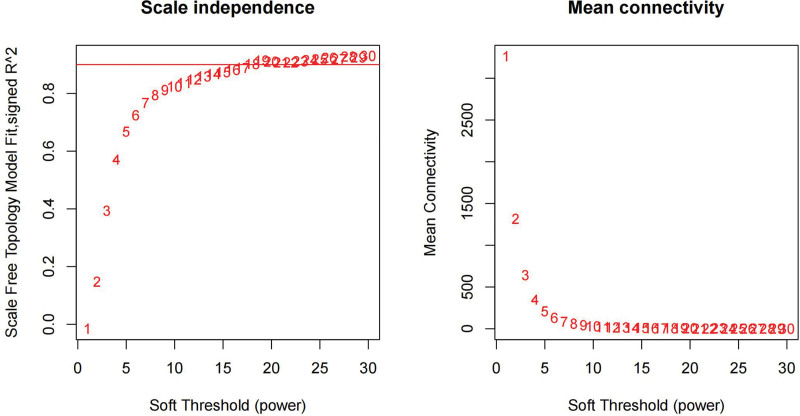
Scale independence.

**Figure 6.**
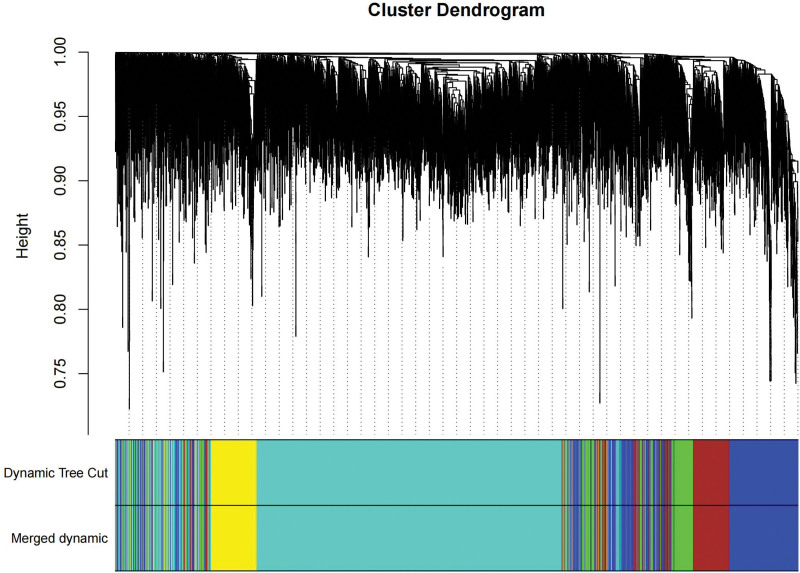
The merging of similar modules.

**Figure 7.**
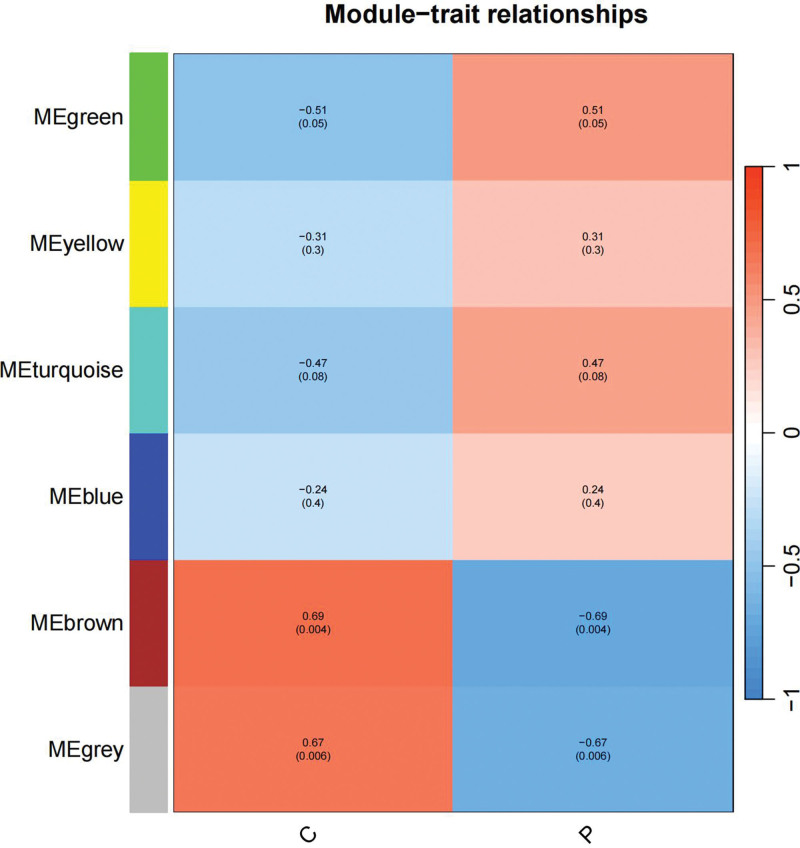
Module-trait relationships.

**Figure 8.**
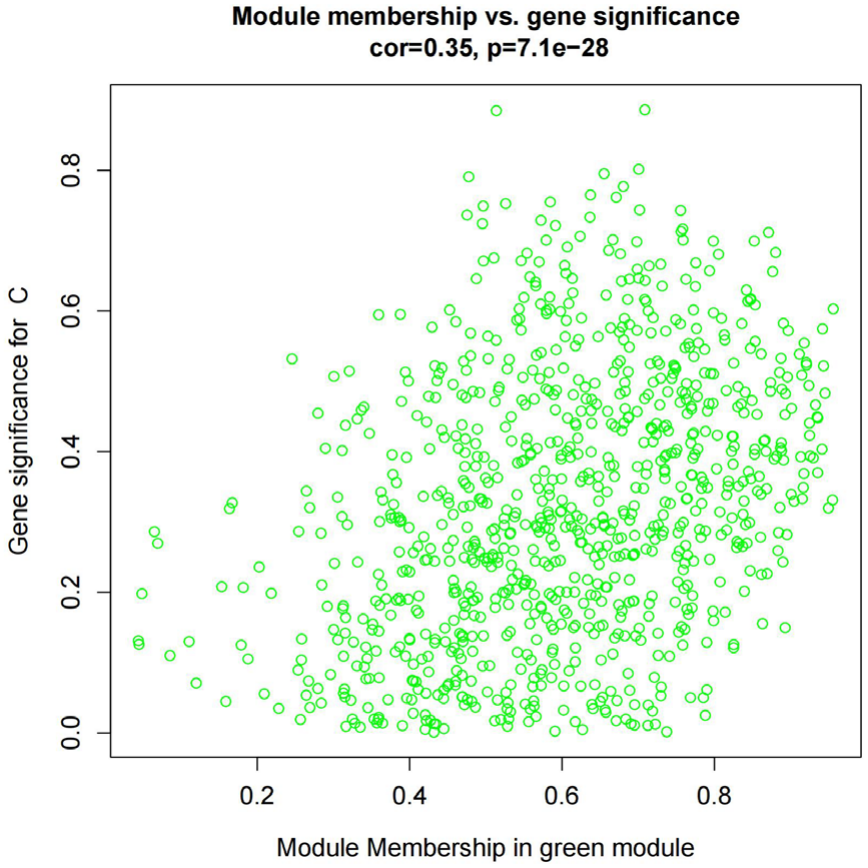
Module membership in green module.

**Figure 9.**
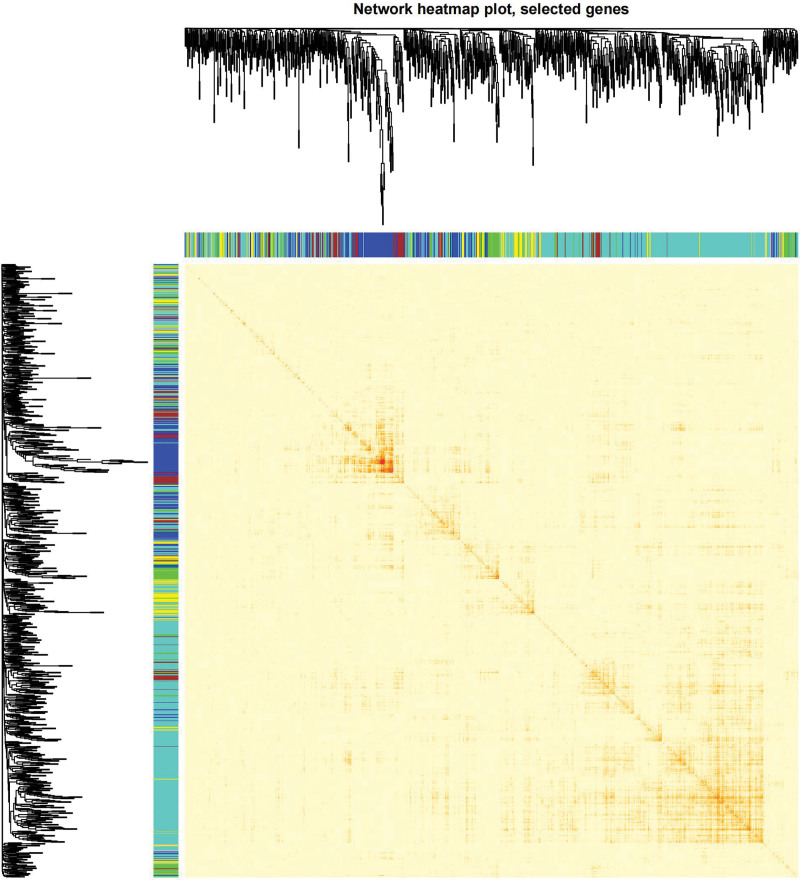
Clustering dendrogram of module feature genes.

**Figure 10.**
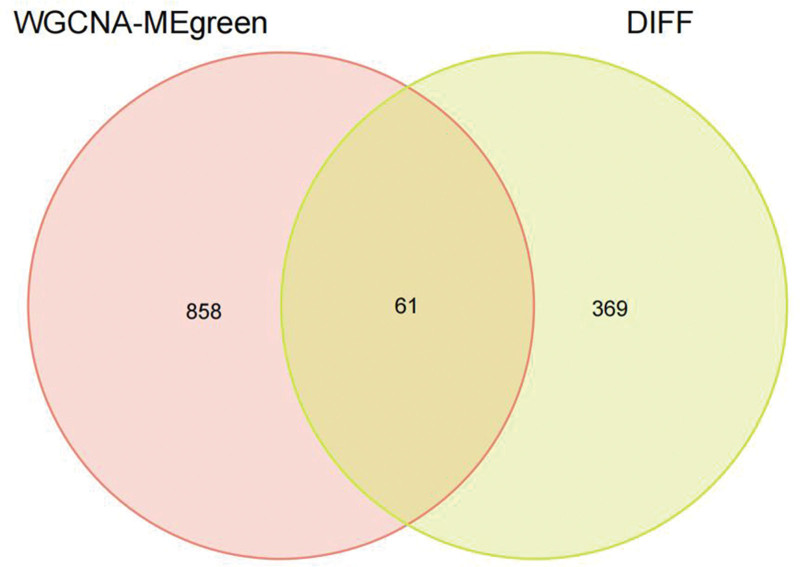
Co-expressed genes.

We constructed a sample clustering tree (Fig. 4) and obtained the soft threshold of 13 (Fig. 5). Merging the similar modules (Fig. 6), we obtained the associations of module-trait of 12 modules (Fig. 7). MEgreen was positively correlated with the experimental group and picked as a key module (Fig. 8). (*R* = 0.51; *P* = .05) and 920 genes were screened in the green module (Table S2, http://links.lww.com/MD/L700). The study of transcriptional correlations within modules confirmed the effectiveness of how the modules were delineated, showing no significant relationships between different modules (Fig. 9). We intersected the DEGs and genes from the MEgreen module identified using WGCNA and obtained 61 co-expressed genes. (Fig. 10).

### 3.3. Enrichment analysis

The functional analysis of co-expressed genes identified the related pathways of DN and analyzed the possible roles of these genes in the related pathways. After integrating relevant data sources, the pathways were mapped, as shown in KEGG and GO.

**Figure 11.**
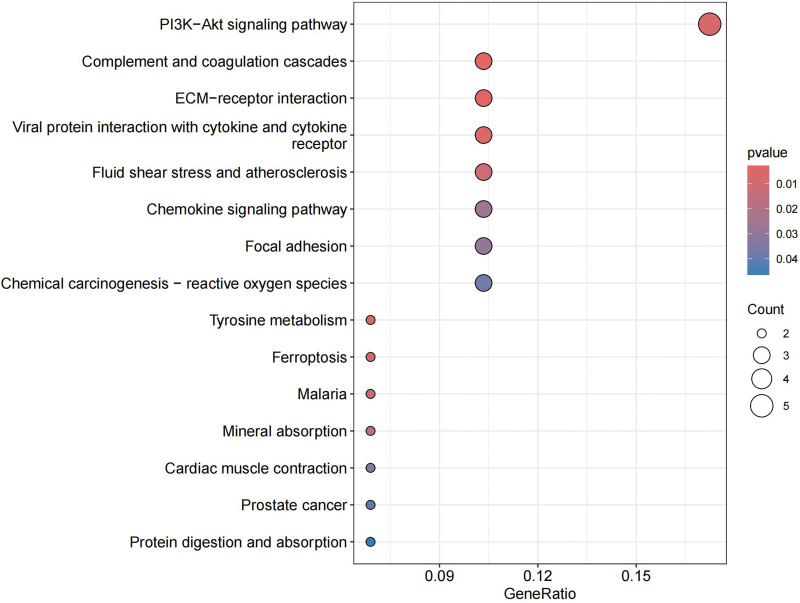
KEGG. KEGG = Kyoto encyclopedia of genes and genomes.

**Figure 12.**
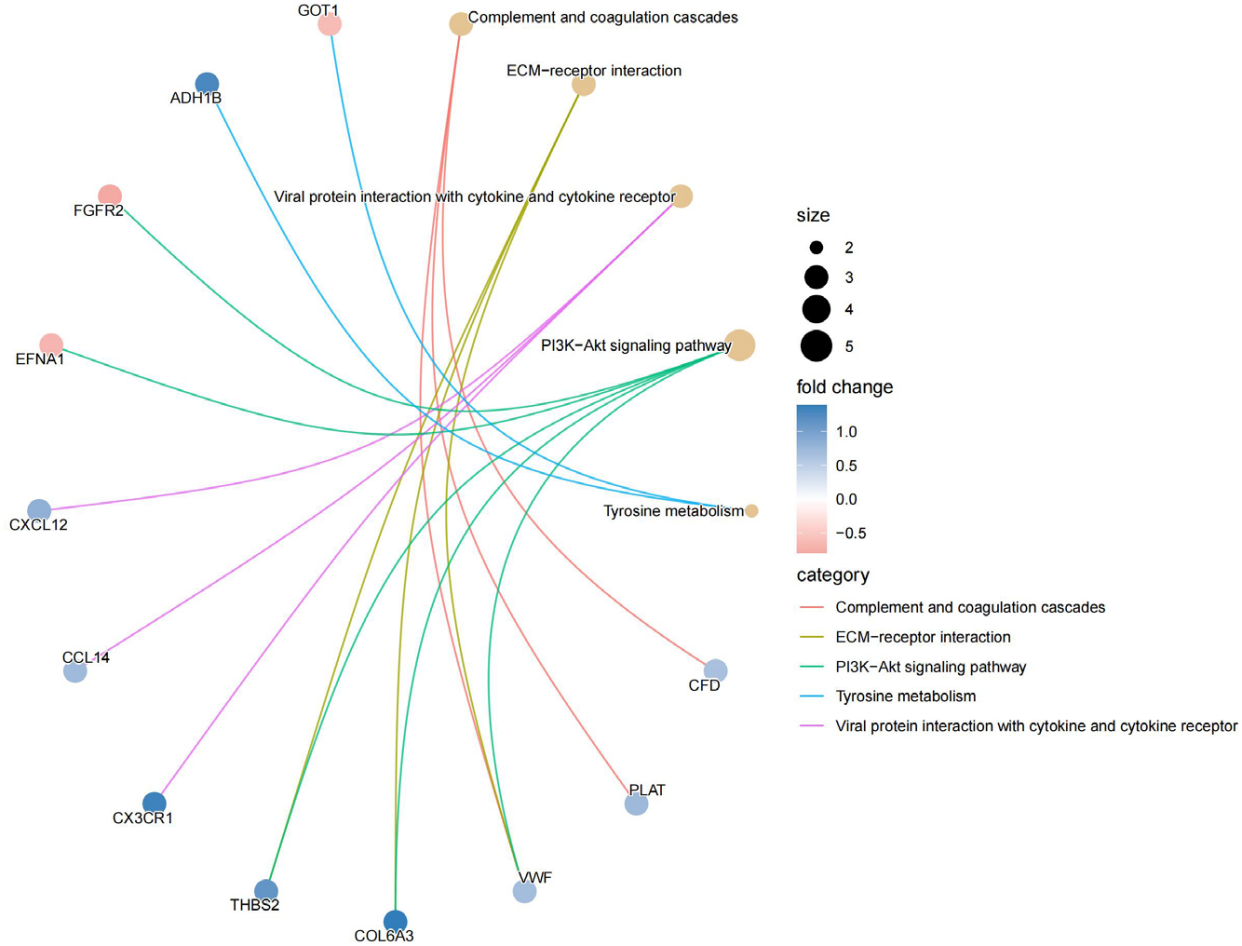
KEGG cnetplot. KEGG = Kyoto encyclopedia of genes and genomes.

**Figure 13.**
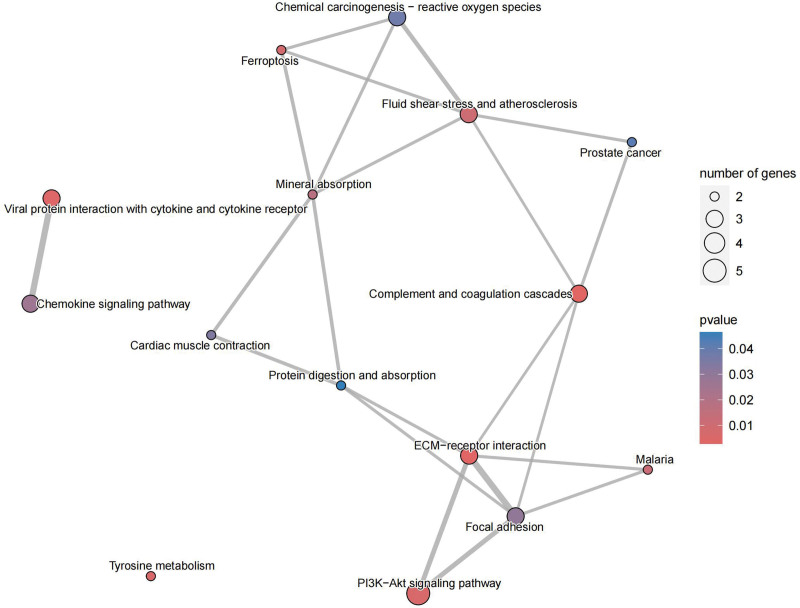
KEGG net. KEGG = Kyoto encyclopedia of genes and genomes.

**Figure 14.**
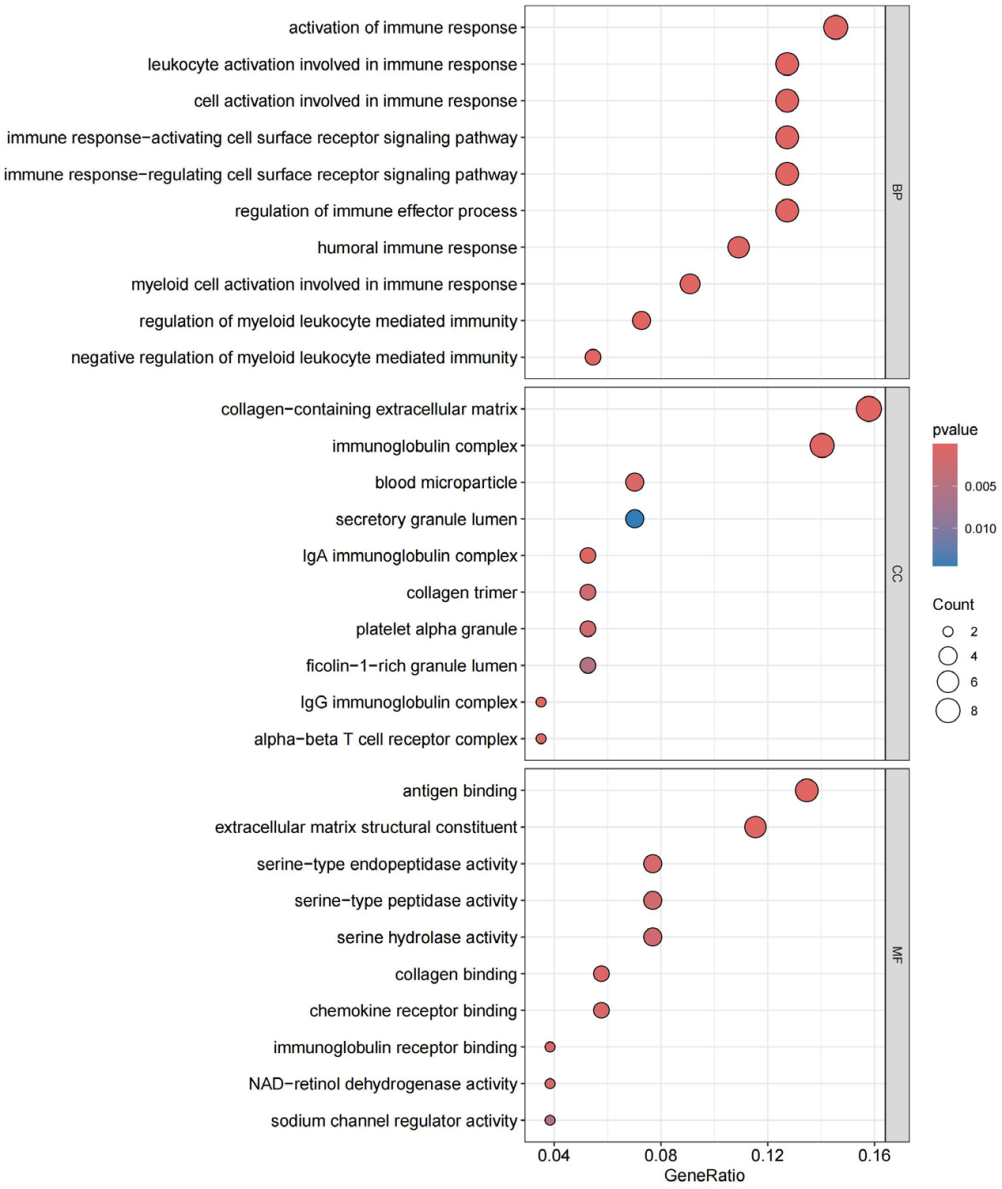
GO. GO = gene ontology.

In KEGG, these genes are closely related to PI3K-Akt signaling pathway, complement and coagulation cascades, ECM-receptor interaction, and chemokine signaling pathway (Fig. 11). The relationship of these functions to genes can also be clearly seen (Fig. 12). In addition, there are some connections between these pathways or functions, and we can thus see more intuitively which one is at the core (Fig. 13). GO analysis includes BP, cellular component (CC), and molecular function (Fig. 14). BP mainly included negative regulation of myeloid leukocyte mediated immunity, myeloid cell activation involved in immune response, and immune response. CC mainly included some multiple complexes. Such as immunoglobulin, IgA immunoglobulin, collagen-containing extracellular, IgG immunoglobulin, and alpha-beta T cell receptor. Molecular function also includes multiple bindings and activities. For example, antigen, immunoglobulin receptor, collagen, serine-type endopeptidase, and serine hydrolase.

### 3.4. Identification of hub genes

**Figure 15.**
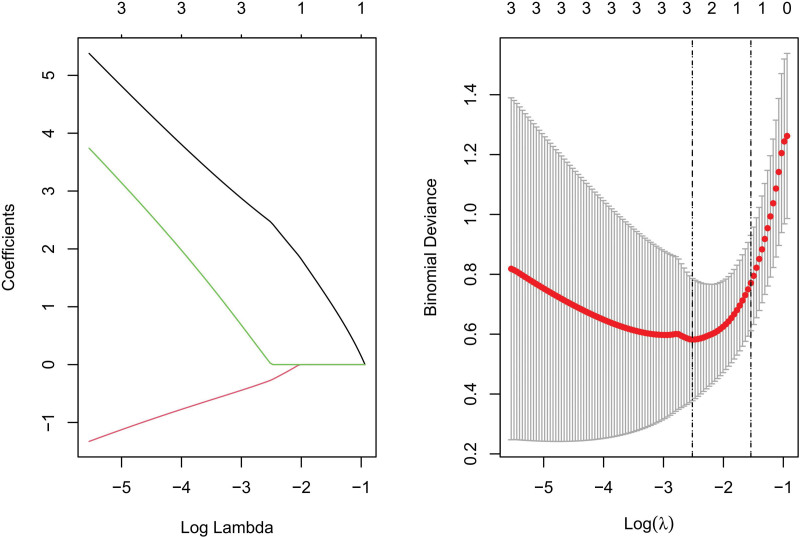
LASSO. LASSO = lasso regression algorithm.

**Figure 16.**
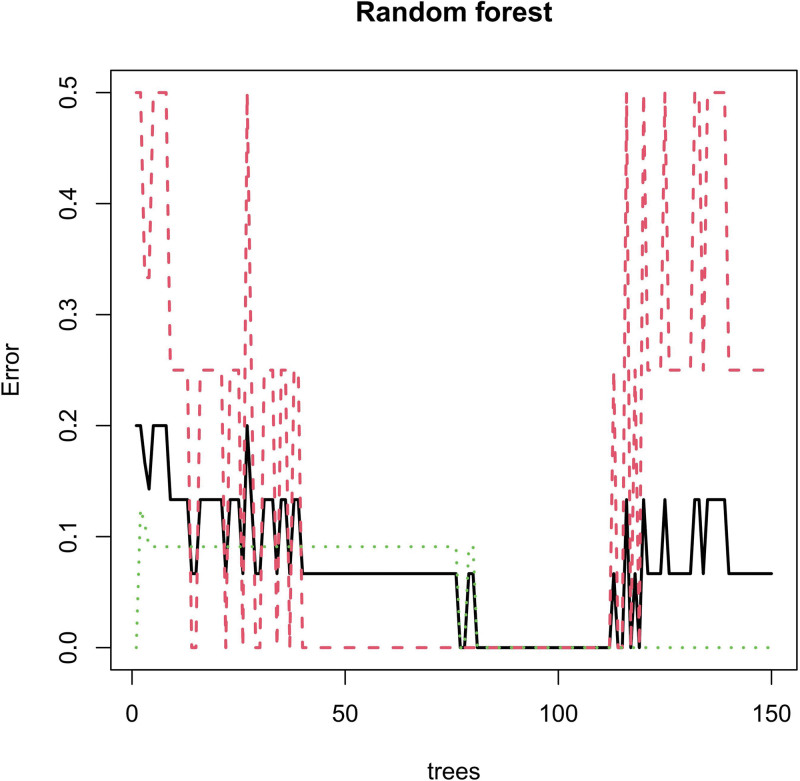
Random forest.

**Figure 17.**
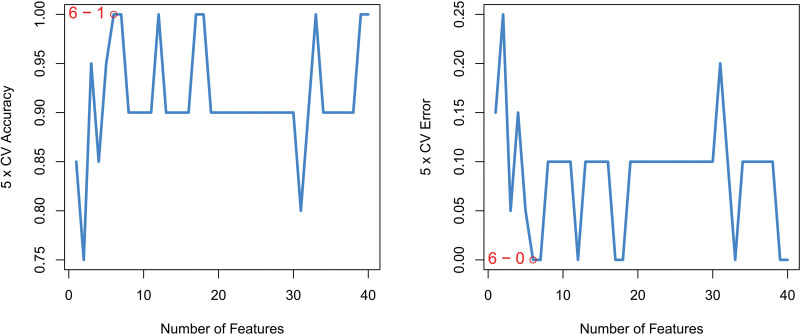
SVM-REF. SVM-REF = support vector machine-recursive feature elimination.

**Figure 18.**
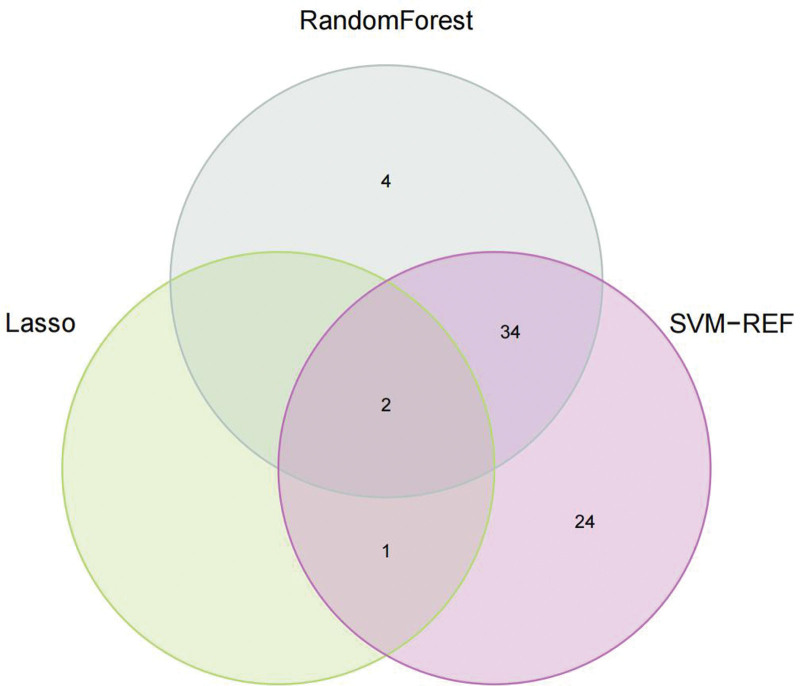
Hub genes.

We conducted Random Forest, LASSO, and SVM-REF screening in order to further identify the hub genes. Through LASSO screening, we obtained 3 genes (Fig. 15). Through Random Forest screening, we obtained 41 genes (Fig. 16). Through SVM-REF screening, we obtained 61 genes (Fig. 17). After the intersection of the genes screened using Random Forest, LASSO, and SVM-REF, 2 hub genes were obtained. They were VWF and DNASE1L3 (Fig. 18).

### 3.5. Verification of hub genes

**Figure 19.**
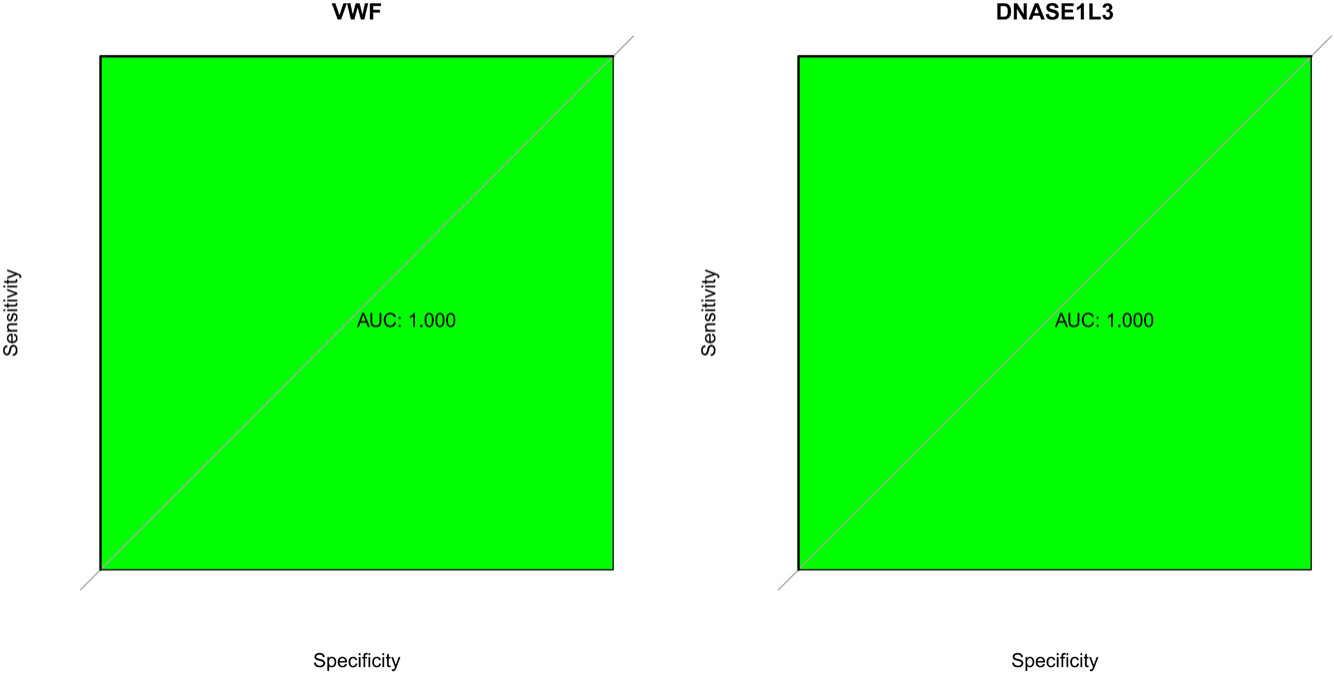
ROC of hub genes. ROC = receiver operating characteristic.

**Figure 20.**
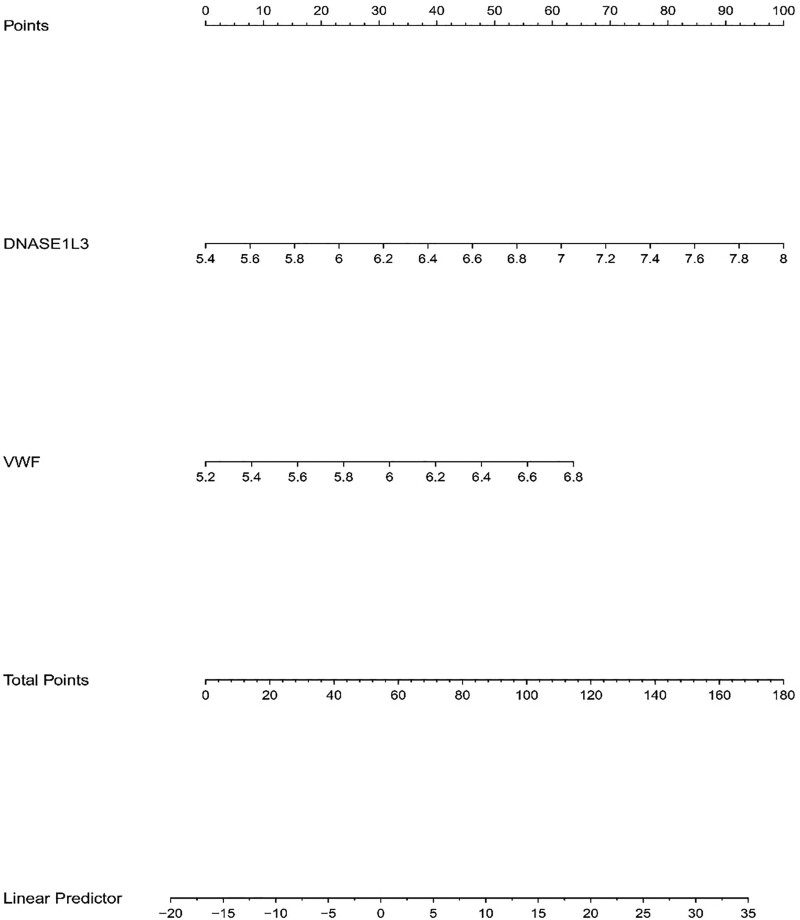
Prediction model of nomogram.

**Figure 21.**
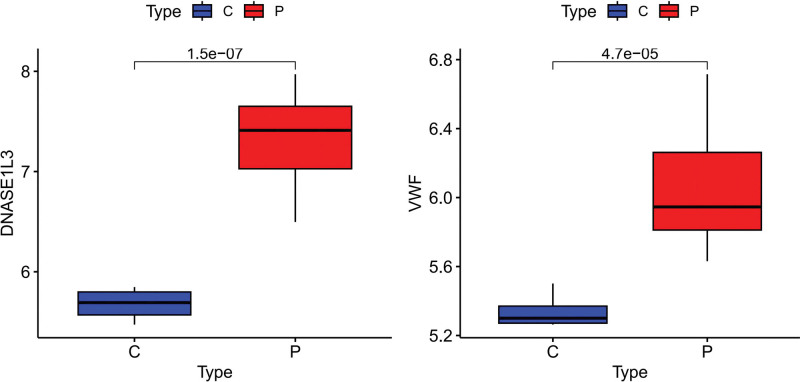
Box plots of hub genes.

**Figure 22.**
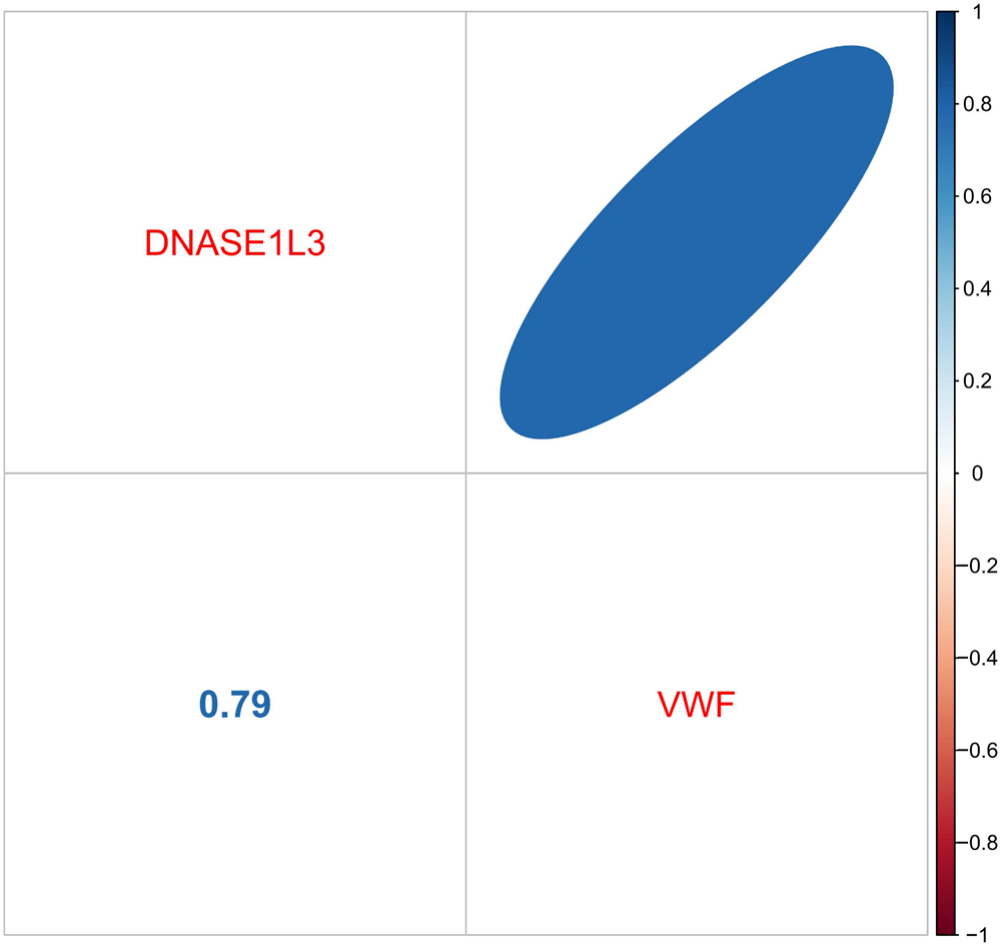
Correlation heatmap of genes.

We assessed the diagnostic potential of 2 specific genes using ROC curves in our dataset, where these genes demonstrated a perfect AUC of 1, indicating high accuracy (Fig. 19). This suggests that our predictive model (Fig. 20) was highly precise. Further analysis confirmed that the expression levels of VWF and DNASE1L3 were notably higher in the experimental samples (Fig. 21). This result was consistent with the results of module-trait relationships and differential analysis. Additionally, a correlation heatmap analysis of key genes (Fig. 22) revealed strong similarity between VWF and DNASE1L3 This aligns with our earlier findings regarding DEGs. This also enhances the precision of our study.

### 3.6. ssGSEA

**Figure 23.**
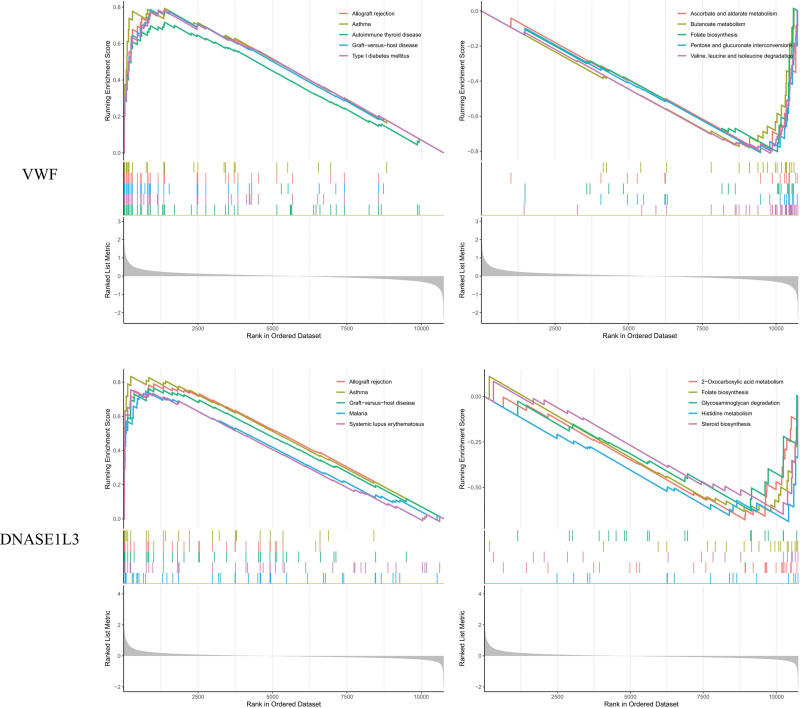
ssGSEA. ssGSEA = single sample gene set enrichment analysis.

**Figure 24.**
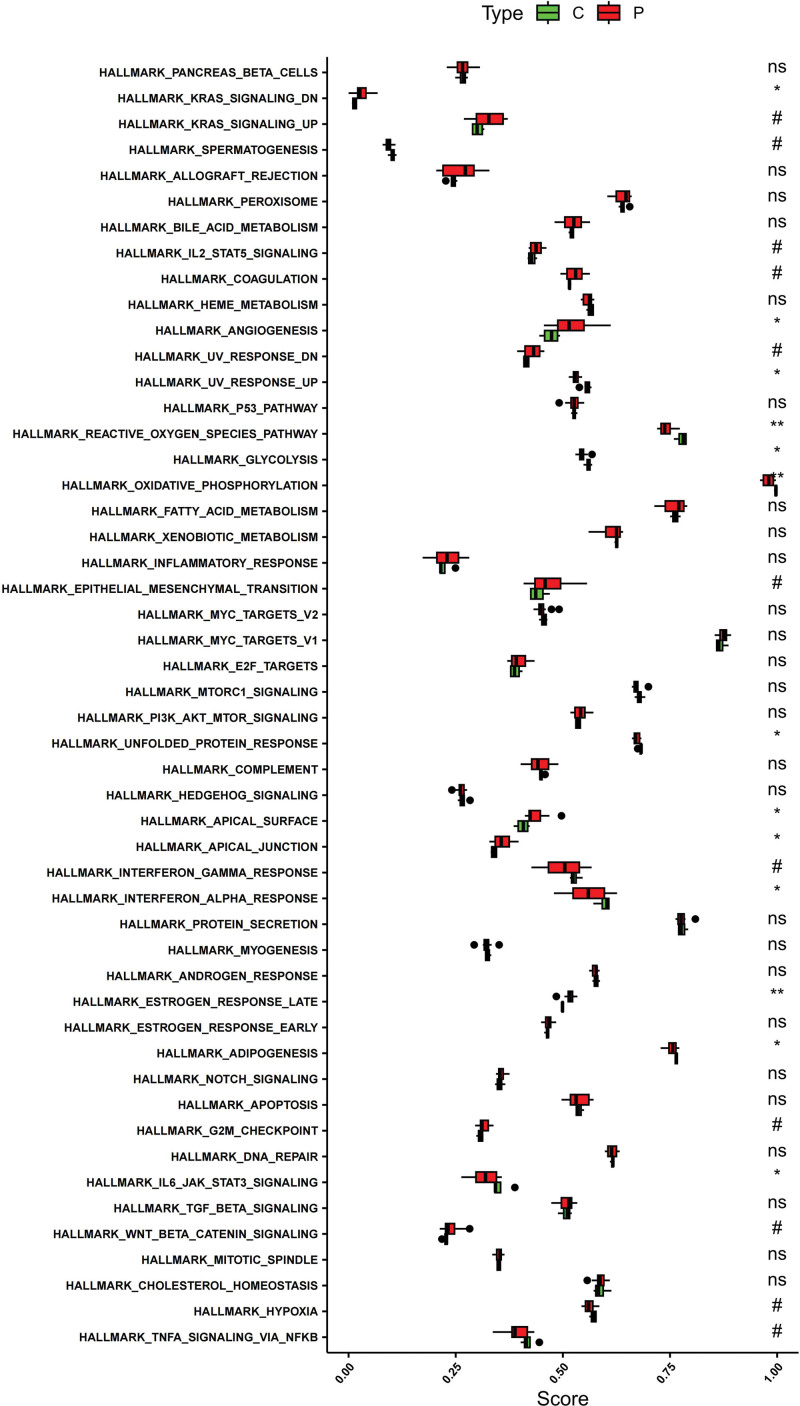
Difference comparison of ssGSEA. ssGSEA = single sample gene set enrichment analysis.

ssGSEA (single-sample Gene Set Enrichment Analysis) is a method used to assess the presence and activity of specific gene sets in individual samples, particularly in transcriptomic data. It ranks genes by expression levels and calculates enrichment scores for gene sets, aiding in identifying biomarkers and understanding biological pathways in disease and treatment studies. In ssGSEA analysis, these genes were highly functionally similar and were all related to pathways and functions such as Chemokine, NF-kappa B, and Wnt signaling pathway (Fig. 23, Table S3 and S4; http://links.lww.com/MD/L701; http://links.lww.com/MD/L703). We also compared the differential expression of hub genes in the experimental and control groups (Fig. 24). In the HALLMARK signaling pathway, KRAS SIGNALING DN, ANGIOGENESIS, UV RESPONSE UP and REACTIVE OXYGEN SPECIES PATHWAY were different between the 2 groups of samples.

## 4. Discussion

In the differential analysis, Module-trait relationships, Machine Learning Algorithms, and Box plots, both VWF and DNASE1L3 showed significant expression in the DN group, suggesting a high likelihood that VWF and DNASE1L3 are pathogenic genes for DN.

In patients with DM, increased levels of VWF have been linked to a higher risk of complications such as cardiovascular diseases and DN. As DN progresses, a surge in VWF can intensify microvascular damage, a key feature of this condition.^[15]^ VWF is a large, multimeric protein produced by endothelial cells that is essential for blood coagulation. High blood sugar levels over extended periods in diabetics can damage endothelial cells, leading to increased VWF production.^[16]^

Research indicates that an imbalance in VWF is associated with elevated hypercoagulability and a greater risk of atherosclerotic cardiovascular disease in patients with DN.^[17]^ Factors like renal dysfunction and inflammation, or their combination, may trigger this imbalance. In diabetic patients, hypercoagulability and increased inflammation could lead to endothelial dysfunction and higher VWF levels,^[18]^ potentially causing renal injury.^[19]^ The development of nephropathy could further worsen hypercoagulability and inflammation, increasing VWF levels and contributing to atherosclerotic cardiovascular disease. This suggests that VWF imbalance plays a crucial role in the development of renal and cardiovascular complications in DM.

A study^[20]^ using mouse models of cancer and hyperglycemia found that high blood sugar levels significantly increase metastasis by enhancing the adhesiveness of endothelial cells, facilitating tumor cell adhesion and migration. VWF, upregulated in endothelial cells due to oxidative stress from hyperglycemia, is critical in this process, making it a potential target for managing hyperglycemia-induced tumor metastasis.

Further research^[21]^ using high-energy sequencing and preliminary in vitro experiments identified miR-149-5p and TNF-α as a differentially expressed mRNA/miRNA pair in Type 2 Diabetes Mellitus (T2DM) with vascular injury.^[22]^ This study showed that miR-149-5p directly targets TNF-α, and increasing miR-149-5p levels can alleviate high glucose-induced dysfunction in endothelial cells.^[23]^ This improvement involved a decrease in ET-1, VWF, and ICAM-1 levels, increased NO production, and enhanced eNOS expression. Additionally, miR-149-5p was found to mitigate cell injury and reduce apoptosis by normalizing endoplasmic reticulum stress markers increased by high glucose levels. This research sheds light on the relationship between VWF and endothelial cells in T2DM, offering insights for developing therapies for vascular complications associated with the condition.

Elevated levels of VWF in DN not only facilitate blood coagulation but also intensify inflammation and thrombus formation.^[24]^ This increase in VWF, particularly in the kidneys, can lead to microvascular damage, contributing to thrombotic microangiopathy,^[25]^ resulting in renal ischemia and cellular injury. Over time, this can exacerbate the severity of DN by causing a decline in renal function. The excessive VWF contributes to platelet aggregation within damaged microvessels,^[26]^ forming thrombi that can further impair microvascular function and limit blood flow. This thrombosis in the kidneys hinders filtration efficiency, potentially leading to tissue damage and worsening the progression of DN. Additionally, the combined effects of VWF and thrombus formation may promote inflammatory responses and fibrosis, leading to scar tissue formation in the kidneys,^[27]^ which further aggravates the condition.

In DN patients, kidney damage, which intensifies due to the progression of the disease, is likely to cause an increased rate of cell death, especially in diabetic kidneys. This process results in a rise of kidney-released cell-free DNA (cfDNA) in the patients’ plasma, characterized by unique kidney-specific digestion signals. Research^[28]^ involving the analysis of RNA-seq data from paired blood cells and kidney tissues of 18 individuals,^[29]^ sourced from the Genotype-Tissue Expression database, was conducted to validate this hypothesis. This research revealed that the DNASE1L3 gene expression level in kidney tissue is markedly higher—about 40 times—compared to that in blood cells. Therefore, the distinctive cfDNA end motifs found in DN patients can be primarily attributed to the cellular nuclease activity in their plasma. These insights significantly contribute to understanding the generation mechanisms of cfDNA in the plasma of DN patients,^[30]^ underscoring the crucial role of DNASE1L3 in this context.

## 5. Conclusion

In summary, our research on DN markers stands out due to its use of diverse analytical techniques, including WGCNA, LASSO, Random Forest, and SVM-RFF, for identifying genes related to the disease. These methods are instrumental in pinpointing key genes that play a role in the onset and advancement of the disease.

In this study, we used a variety of methods that play an important role in clinical trials and medical research: WGCNA is used to analyze gene expression data, help identify potential biomarkers and disease subtypes, and provide clues to individualized treatment. KEGG and GO are commonly used for pathway and functional analysis to help understand genes and disease-related pathways and guide drug development and treatment strategies. SVM-RFE and LASSO play a role in feature selection for identifying critical features. Random Forest is suitable for large datasets, provides highly accurate classification and prediction, and is helpful for individualized medical decision-making, such as disease risk assessment.

SVM-RFE, LASSO, and Random Forest we used are innovative machine learning techniques. SVM-RFE innovation is in its recursive feature elimination, enhancing SVM applications in pattern recognition and bioinformatics. LASSO uniqueness is in penalizing coefficient sizes, aiding in feature selection and regularization, and enabling sparse solutions. Its path algorithm is also computationally efficient. Random Forest innovation lies in its ensemble learning approach, combining multiple decision trees to improve model performance and introducing randomness to reduce overfitting and enhance diversity and robustness. These techniques significantly contribute to advancements in machine learning, particularly in feature selection, regularization, and ensemble methods.

However, our study is not without its limitations. Firstly, there are inherent constraints that hinder the experimental validation of our findings. Secondly, the results, derived from a specific dataset, may not be entirely applicable to the broader spectrum of the disease. Further research is essential to accurately determine the precise molecular mechanisms and functional pathways of these proteins in the context of DN. This entails a deeper exploration of how these proteins interact at the molecular level and the specific roles they play in the progression and development of DN. Understanding these aspects could provide critical insights into the underlying processes of the disease, paving the way for more effective treatments and management strategies.

## Author contributions

**Conceptualization:** Baihan Dong.

**Methodology:** Xiaona Liu.

**Supervision:** Siming Yu.

**Validation:** Siming Yu.

**Writing – original draft:** Baihan Dong.

**Writing – review & editing:** Baihan Dong.

## Supplementary Material








